# The clinical value of aberrant epigenetic changes of DNA damage repair genes in human cancer

**DOI:** 10.18632/oncotarget.7949

**Published:** 2016-03-07

**Authors:** Dan Gao, James G. Herman, Mingzhou Guo

**Affiliations:** ^1^ Department of Gastroenterology and Hepatology, Chinese PLA General Hospital, Beijing, China; ^2^ Medical College of NanKai University, Tianjin, China; ^3^ The Hillman Cancer Center, University of Pittsburgh Cancer Institute, Pittsburgh, PA, USA

**Keywords:** DNA methylation, DNA damage repair, MGMT, synthetic lethality, PARP inhibitor

## Abstract

The stability and integrity of the human genome are maintained by the DNA damage repair (DDR) system. Unrepaired DNA damage is a major source of potentially mutagenic lesions that drive carcinogenesis. In addition to gene mutation, DNA methylation occurs more frequently in DDR genes in human cancer. Thus, DNA methylation may play more important roles in DNA damage repair genes to drive carcinogenesis. Aberrant methylation patterns in DNA damage repair genes may serve as predictive, diagnostic, prognostic and chemosensitive markers of human cancer. MGMT methylation is a marker for poor prognosis in human glioma, while, MGMT methylation is a sensitive marker of glioma cells to alkylating agents. Aberrant epigenetic changes in DNA damage repair genes may serve as therapeutic targets. Treatment of MLH1-methylated colon cancer cell lines with the demethylating agent 5′-aza-2′-deoxycytidine induces the expression of MLH1 and sensitizes cancer cells to 5-fluorouracil. Synthetic lethality is a more exciting approach in patients with DDR defects. PARP inhibitors are the most effective anticancer reagents in BRCA-deficient cancer cells.

## INTRODUCTION

**Figure 1:**
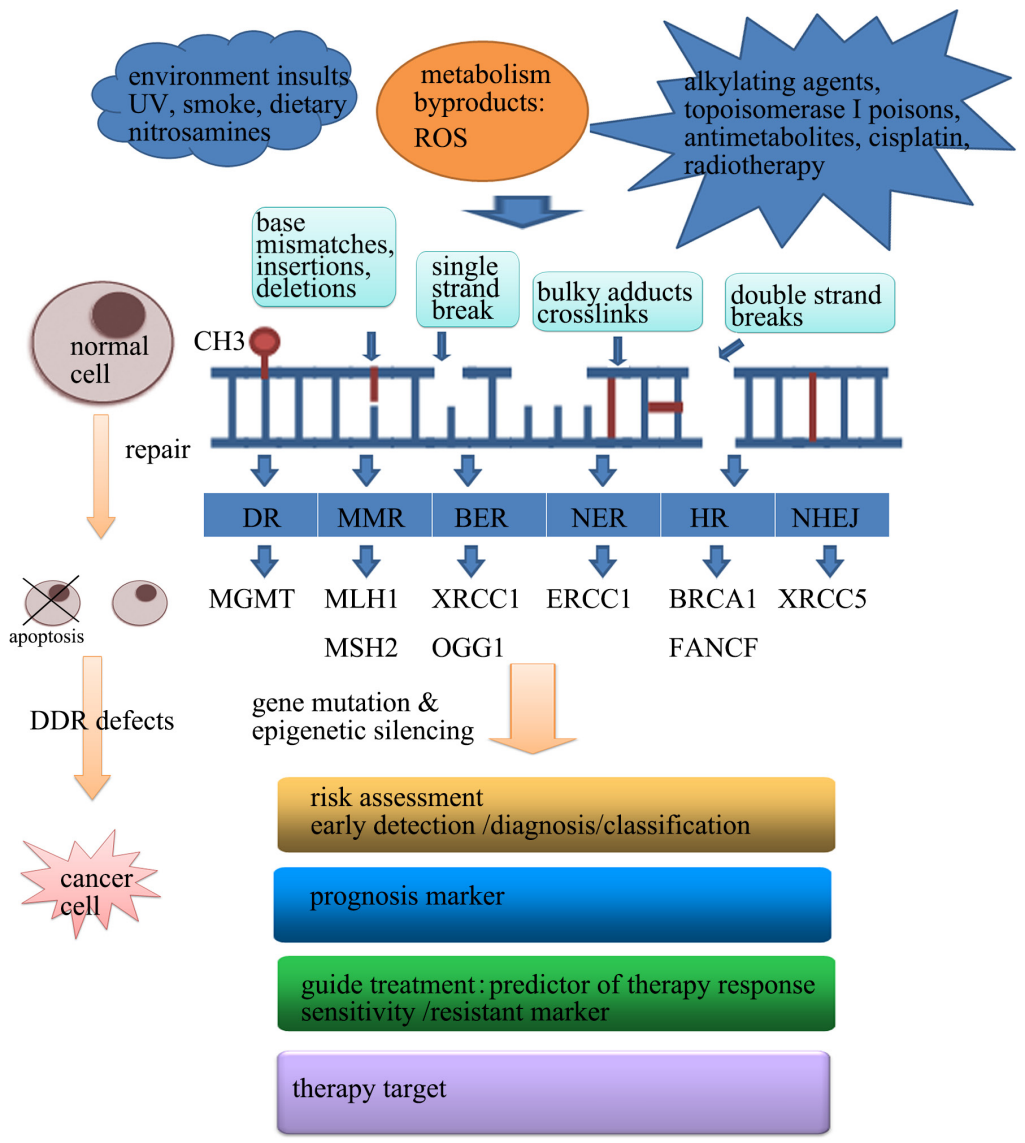
The application of abnormal epigenetic changes of DDR in human cancer

The integrity of the DNA in each cell is continually challenged by hundreds of thousands of insults each day that can alter the sequence or chemical composition of the DNA [1]. These changes include single-strand or double-strand DNA breaks, base damage, bulky adducts, intrastrand and interstrand cross-links and breakdown of the replication fork [1]. To limit severe impacts of DNA damage and replication errors, cells have evolved a plethora of molecular processes that maintain the integrity of the genome [2]. Once DNA is damaged, it can be repaired by at least one of the six major DNA damage repair (DDR) pathways: direct repair, mismatch repair (MMR), base excision repair (BER), nucleotide excision repair (NER), non-homologous end joining (NHEJ) and homologous recombination (HR) [3, 4] (Figure 1). Loss of efficiency of one or more DNA repair pathways can accelerate the rate of accumulation of additional mutations by 100–1,000 times [5]. Unrepaired DNA damage is a major source of potentially mutagenic lesions that drive carcinogenesis [5, 6].

In patients with hereditary non-polyposis colorectal cancer (HNPCC), germ-line defects in mismatch-repair genes (primarily MLH1 and MSH2) confer a lifetime risk of colorectal cancer (CRC) of approximately 80% [7]. Germline mutations in MSH2 and MLH1 account for approximately 60% of HNPCC, while nearly one-third of HNPCC patients do not show MMR gene mutations, which may be attribute to epigenetic silencing [8–11]. Somatic inactivation of the mismatch-repair gene MLH1 occurs in approximately 15% of patients with sporadic colorectal cancers by promoter region methylation in bialleles [12–14]. Heterozygous germline mutations in breast cancer susceptibility gene 1/2 (BRCA1/2) are responsible for a large fraction of hereditary breast cancers. The risk of developing breast cancer by age 70 is 50–70% for BRCA1/2 mutation carriers [6, 15, 16]. While BRCA1 and BRCA2 mutations affect a minority of breast cancer patients (fewer than 5%), BRCA1 and BRCA2 were silenced by promoter region hypermethylation in 9% and 2% of sporadic breast cancer, respectively [17, 18]. O6-methylguanine-DNA methyltransferase (MGMT) is mutated in 17.5% and methylated in 44% of human esophageal squamous cell carcinoma [19, 20]. Thus, aberrant epigenetic changes may play more important roles than gene mutations in DNA damage repair genes to drive carcinogenesis. This review is mainly focused on the role of epigenetics in DNA damage repair genes.

## EPIGENETIC CHANGES OF DNA DAMAGE REPAIR GENES IN HUMAN CANCERS

### Direct repair genes

The simplest form of DNA repair is the direct reversal of the lesion [21]. MGMT removes alkyl groups from O6-methylguanine that are formed as a result of erroneous methylation by S-adenosylmethionine, and it also removes other alkylations at the O6-position of guanine that are induced by dietary nitrosamines or chemotherapy agents such as temozolomide (TMZ), dacarbazine (DTIC) and carmustine (BCNU) [22, 23]. Unrepaired O6-methylguanine gives rise to O6-methylguanine/thymine mispairing, which is recognized and excised by the DNA MMR system during DNA replication [24, 25]. If O6-methylguanine is not repaired by MGMT and MMR, G:C to A:T transition mutations will lead to carcinogenesis when they occur in cancer-related genes, such as K-ras and p53 [26, 27]. MGMT is frequently methylated in various tumors, including gliomas (40%) [28], diffuse large B-cell lymphoma (36%) [29], colorectal cancer (46%) [30], gastric cancer (36.8%) [31], non-small cell lung cancer (21%) [32], esophageal cancer (44%) [19] and head and neck cancers (60.8%) [33].

### Base excision repair pathway

The most versatile of the excision repair systems, BER, is required to repair DNA single-strand breaks and deamination, oxidation, and alkylation-induced DNA base damage that may result from chemotherapy (alkylating agents, topoisomerase I poisons, antimetabolites), radiotherapy, environmental exposure (nitrosamines, ultraviolet light, smoke) or endogenous byproducts of cellular metabolism (reactive oxygen species) [34].

The 8-oxo-G DNA lesion is one of the best-studied types of oxidative DNA damage caused by reactive oxygen species (ROS), and it is weakly cytotoxic but mostly mutagenic [34]. Because 8-oxodG DNA lesions do not block DNA replication, they lead to mispairing with A and result in GC to TA transversions, the most predominant somatic mutations in lung, breast, ovarian, gastric and colorectal cancers [35]. The main components of the BER pathway are glycosylases, endonucleases, DNA polymerases and DNA ligases, with poly(ADP-ribose) polymerase 1 (PARP1) and PARP2 facilitating the process [36]. Mutations and single nucleotide polymorphisms (SNPs) in BER genes are associated with numerous cancers, such as UNG2 (uracil DNA glycosylase) mutations in glioblastoma [37], MED1 (mediator complex subunit 1) mutations in colorectal cancer [38], and OGG1 (8-oxoguanine DNA glycosylase) mutations in lung cancer [39]. The X-ray repair cross-complementing gene 1 (XRCC1) polymorphism, Arg399Gln, increased the occurrence of p53 mutations in human lung cancer [40]. Aberrant methylation of BER genes were found in a variety of cancers. XRCC1 was methylated in 76.4% of human gastric cancers [41]. Aberrant DNA methylation of the TDG (thymine DNA glycosylase) gene is reported in multiple myeloma cells and is related to genomic instability in multiple myeloma [42]. Promoter region hypermethylation of MED1 is reported frequently in colorectal and ovarian cancers [43]. OGG1 is found to be methylated in breast and thyroid cancers [44, 45].

The Werner syndrome gene (WRN), a RecQ family member with both helicase and exonuclease activities, also participates in base excision repair [46, 47]. Germline mutations in WRN are the cause of Werner syndrome, an autosomal recessive disorder characterized by premature aging, genomic instability, and predisposition to cancer [48]. However, somatic mutations of WRN have not been described in sporadic neoplasms [47]. A study in colorectal cancer showed that epigenetic inactivation of WRN leads to loss of WRN-associated exonuclease activity and increased chromosomal instability and apoptosis induced by topoisomerase inhibitors [48]. Promoter region methylation of the WRN gene has been reported frequently in colorectal (37.9%), non-small cell lung (37.5%), gastric (25%), prostate (20%), breast (17.2%), and thyroid (12.5%) cancers, chondro-sarcomas (33.3%), and non-Hodgkin lymphoma (23.7%) [47].

### Nucleotide excision repair genes

The NER genes recognize and repair bulky DNA damage that involves more than one nucleotide, including adducts induced by chemical carcinogens, such as tobacco smoke, platinum compounds and photoproducts, or dimers induced by ultraviolent (UV) light [49]. NER abnormalities are present in several rare recessive photosensitive syndromes, including xeroderma pigmentosum (XP), Cockayne syndrome (CS) and trichothiodystrophy (TTD) [50]. XP is the first human disease that was clearly associated with defects in DNA repair. It is a rare autosomal recessive disease caused by biallelic inactivating mutations in genes involved in nucleotide excision repair [51]. Patients with mutations in XP genes are extremely sensitive to UV and have extraordinarily high incidences of non-melanoma skin cancer and melanoma, as well as other solid tumors [49]. In 158 cases of lung cancer samples, promoter region methylation of the XPC (xeroderma pigmentosum group C) gene was found in 53 cases (34%). Interestingly, XPC methylation was more common in nonsmokers (39/94, 41%) than in smokers (14/64, 22%) [52]. The XPC gene was methylated in 32.5% of bladder cancers [53]. ERCC1 (excision repair cross-complementation group 1) was methylated in 37.5% of gliomas [54]. hHR23B (RAD23 homolog B) was methylated in multiple myeloma cells [55]. XPG was methylated in 20% of ovarian cancers [56].

### Mismatch repair genes

The MMR system recognizes base–base mismatches and insertion or deletion loops (IDLs) in double-stranded DNA, and it degrades the error-containing region of the newly synthesized strand, allowing the polymerase to correctly re-synthesize the second strand according to the template sequence [48]. The human MMR system includes the MLH1, MLH3, MSH2, MSH6, PMS1 and PMS2 genes [57, 58]. Defective MMR increases mutation rates up to 1,000-fold and leads to microsatellite instability (MSI) resulting in carcinogenesis [22]. The MSI status of a tumor can be detected by five microsatellite markers which are composed of two mononucleotide repeats (BAT25, BAT26) and three dinucleotide repeats (D2S123, D5S346 and D17S250) [59]. If two or more of the five loci show instability, the tumor is MSI-H; if only one locus is unstable, the tumor is MSI-L; and if all five loci are stable, the tumor is MSS [60]. MSI-H is known to occur in more than 90% of HNPCC patients [61]. Inactivation of germline mutations within at least one of four mismatch repair genes (MLH1, MSH2, MSH6, and PMS2) can be found in approximately 70% of cases, and 95% of the mutations occur in hMSH2 or hMLH1 [62]. Loss of expression of the MLH1 or MSH2 genes is approximately 100% associated with the MSI-H phenotype [60]. In population-based studies, the prevalence of MSI among CRCs is approximately 15% [51, 63, 64]. Germline MMR mutations that give rise to HNPCC account for ~3% of all CRCs [63].

In contrast to HNPCC, sporadic cancers are rarely found to have mutations in the MLH1 or MSH2 genes. Researchers have confirmed that promoter-region methylation accounts for 80–90% of MLH1 inactivation in sporadic MSI-H colorectal cancer [60]. In a cohort study that included 268 cases of CRC, 46 cases were MSH2-deficient HNPCC, 15 were cases of sporadic MSI CRCs, and 207 were cases of sporadic MSS CRCs. The study found that 80% of MSH2-deficient HNPCC patients harbored germline mutations in MSH2 [65]. However, MSH2 was methylated in 24% (11/46) of MSH2-deficient HNPCC. Moreover, 63% (7/11) of MSH2 methylated CRCs had a simultaneous pathogenic germline MSH2 mutation, but no methylation was found in matched normal tissues, suggesting that methylation may be the required “second hit” in these tumors [65]. MSH2 was not methylated in any of the sporadic CRCs. Promoter region hypermethylation of MLH1 is also found in other sporadic tumors, including adult T-cell leukemia/lymphoma (6%) [66], gastric cancer (21.6%) [67], head and neck squamous cell carcinomas (28.6%) [68], non-small cell lung cancer (55.8%) [69], oral squamous cell carcinoma (76%) [70], esophageal squamous cell carcinoma (23%) [71], pancreatic cancer (23%) [72], ovarian cancer (37.5%) [73], and breast cancer (31.3%) [74].

In addition to somatic methylation, germline epimutation, a condition in which a person has hypermethylation of one allele of MLH1 in somatic cells throughout the body, increased the risk of CRC by 60% for first-degree relatives of MLH1-methylated cases. For second-degree relatives, there was no evidence of an increased CRC risk. Risks were increased for gastric cancer for first and second-degree relatives. The incidence of liver cancer was also increased for first-degree relatives [75].

### DNA double-strand breaks (DSB) repair

DSBs do not occur as frequently as the other lesions listed above, and they are difficult to repair and extremely toxic [76]. Damaging agents emanating from endogenous and environmental sources constantly challenge the integrity of DNA [77]. Almost all human cancers are characterized by genomic instability, which is considered to play a key role in the conversion of a normal cell into a premalignant cell [4, 77]. DSBs are considered to be the most hazardous lesions, since a single unrepaired DSB may trigger cell death, whereas a misrepaired DSB may result in chromosomal rearrangements [77, 78]. Interstrand crosslinks (ICLs) represent the most deleterious lesions produced by chemotherapeutic agents such as mitomycin C (MMC), cisplatin and cyclophosphamide [77]. ICL repair is complex and involves the collaboration of several repair pathways, namely Fanconi anemia, NER, translesion synthesis and homologous recombination [79]. Inhibition of repair by compounds that target factors involved in NHEJ or HR will increase the sensitivity of cancer cells to DSB-inducing anticancer agents. There are numerous proteins involved in DSB repair, and inactivation of DSB repair genes is associated with various tumors. XRCC5 is an important component of NHEJ. Promoter region methylation of XRCC5 was reported in 21% of non-small cell lung cancers (NSCLCs) [80]. Genes of the Fanconi Anemia (FA) pathway also play an important role in repair by homologous recombination. The FA pathway is not constitutively active in normal cells, but it is turned on during the S phase of the cell cycle or following DNA damage [81]. FA is a rare chromosomal instability syndrome characterized by aplastic anemia in childhood, susceptibility to leukemia and cancer, and hypersensitivity of FA cells to interstrand DNA crosslinking agents, such as cisplatin and melphalan [81]. There are thirteen FA genes, and one of these genes is identical to the well known breast cancer susceptibility gene, BRCA2 [82]. FA proteins and another breast/ovarian cancer susceptibility protein, BRCA1, cooperate in a DNA repair pathway, which is required for resistance to DNA interstrand crosslinks [81, 83]. FA patients with biallelic mutations in BRCA1 have not been identified, perhaps because biallelic loss of BRCA1 results in embryonic lethality [81]. BRCA1 appears to play a relatively early role in the regulation and promotion of HR. BRCA1 is phosphorylated in response to DNA DSBs by several kinases and likely acts in DNA damage signal transduction [84]. Germline mutations in the BRCA1 and BRCA2 genes are the most important causes of hereditary breast and ovarian cancer. While there is not a direct procedure to estimate the prevalence of BRCA1/2 mutations in the general population, it is estimated that nearly 50% of the mutations would be in BRCA1 and 50% in BRCA2 [85]. Lifetime cancer risks in BRCA mutation carriers are 60-80% for breast cancer and 20-40% for ovarian cancer [86]. Mutations in BRCA genes cannot account for all cases of hereditary breast and ovarian cancer. The remaining cases can be attributed to the involvement of constitutive epimutations or other cancer susceptibility genes [86]. The FANCD1, FANCN and FANCJ genes have been shown to be breast cancer susceptibility genes [81].

In addition to germline mutations in these genes occurring in FA, somatic mutations and epigenetic silencing of these genes occur in a variety of cancers in the general population (non-FA patients) [87, 88]. FANCF promoter methylation has been detected in 21% of ovarian, 17% of breast, 15% of head and neck, 30% of cervical, 14% of non-small cell lung, and 11.4% of gastric cancers [48, 89]. FANCC was methylated in 42% of head and neck squamous cell carcinoma according to PCR-based methylation-sensitive restriction analysis (MSRA) [90]. FANCC was methylated in 1 of 143 acute myeloid leukemia (AML) cases and 3 of 97 acute lymphoblastic leukemia (ALL) cases, while FANCL was found to be methylated in one ALL sample [91]. BRCA1/BRCA2 mutations are often found in hereditary breast and ovarian cancers, while somatic mutations of BRCA1/BRCA2 are rare in sporadic breast and ovarian cancers [92, 93]. BRCA1 methylation is observed in approximately 11–14% of breast cancers and 5%–31% of ovarian cancers [93]. BRCA1 methylation is also found in gastric (1.4%) [89], colorectal (10.75%) [94], non-small cell lung (18.6%) [95], bladder (12.1%) [96] and pancreatic cancers (46%) [97]. BRCA2 methylation is reported in NSCLC (42%) [80].

### DNA damage repair pathway and cell cycle checkpoints

The cell cycle may be arrested at either the G1/S or the G2/M transition by cell cycle inhibitors. The resulting cell-cycle arrest allows time for DNA repair, thereby preventing genome duplication or cell division in the presence of damaged DNA. Inducing cell-cycle arrest may reduce the efficacy of DNA-damaging agents used in cancer therapy [77]. The checkpoint with forkhead and ring finger domains (CHFR) gene is an early mitotic checkpoint gene that functions as a key player in controlling chromosomal integrity [98]. CHFR methylation is regarded as a docetaxel sensitive marker [67]. Re-expression of CHFR induced G2/M phase arrest and increased resistance to docetaxel in esophageal cancer cells [99]. CHFR is methylated in CRC (24–53%), gastric cancer (35–52%), esophageal cancer (16.3%) and NSCLC (10–40%) [100]. In docetaxel-treated gastric cancer patients, resistance to docetaxel was found in CHFR unmethylated patients and overall survival was longer in the CHFR methylated group compared to the CHFR unmethylated group [100]. Methylation of p16 has been demonstrated to sensitize gastric cancer to 5-fluorouracil therapy [101]. P16 is a cell cycle regulator that binds specifically to CDK4/6 and inhibits the activity of the CDK4/6-cyclin D1 complex, consequently blocking phosphorylation of retinoblastoma. This allows dissociation of the transcription factor E2F and subsequent transit to the S-phase [101, 102]. Therefore, the cell cycle is disrupted by P16 methylation and the cell number in the S-phase is increased. In this situation, the cancer cell easily absorbs 5-fluorouracil into S-phase cells, where it acts as an antimetabolite agent, leading to the inhibition of cancer cell growth [101]. Thus, selective disruption of the G1/S or G2/M checkpoints sensitizes cancer cells to chemotherapy.

## ABERRANT METHYLATION OF DNA DAMAGE REPAIR GENES MAY SERVE AS PREDICTIVE, DIAGNOSTIC AND PROGNOSTIC MARKERS OF HUMAN CANCER

DNA repair pathways are frequently disrupted through genetic and epigenetic mechanisms. MSI and DNA repair gene mutations have been used as diagnostic and prognostic markers in various tumors [1, 103, 104]. Germ-line mutations in MLH1 and MSH2 suggest a very high risk of colorectal cancer [7]. The prognosis is much better in sporadic colorectal cancers with a mismatch repair deficiency [63]. The overall breast cancer and ovarian cancer risks for BRCA1 mutation carriers by age 70 years are 50-70% and 40-50%, respectively [6]. Thus, detection of the susceptible genes may guide the frequency of cancer surveillance and decision making for prophylactic surgery [7].

DNA methylation represents the epigenetic biomarker with the highest translational potential due to its stable nature and reliable detection technologies [105, 106]. Methylation of DDR genes has been detected in various cancers. MGMT is frequently methylated in various tumors [48]. MGMT methylation is a marker of poor prognosis in human glioma, without treatment with an alkylating agent. While, silencing MGMT by promoter region hypermethylation sensitizes glioma cells to alkylating agents [107]. MGMT methylation is more frequent in the margins of lung and colorectal cancer tissue samples than in the most distant sites from the resected specimen [30, 108]. This suggests that MGMT methylation is a potential early detection marker for lung and colorectal cancers. MLH1 was methylated in human esophageal dysplasia and cancer samples, but it was unmethylated in normal esophageal epithelia [71]. MLH1 methylation may serve as an early detection marker for esophageal cancer. MLH1 methylation was reported to be a good prognostic marker in colorectal and pancreatic cancers [63, 72]. CHFR methylation is associated with an increased risk of disease recurrence and poor survival in NSCLC and CRC [100]. The OGG1 gene is frequently methylated in different cancers, and silencing of its expression by promoter region methylation is associated with poor prognosis in breast cancer [109]. XPC methylation was associated with the malignant behavior of bladder cancer and may predict poor prognosis [53].

## ABERRANT EPIGENETIC CHANGES OF DNA DAMAGE REPAIR GENES MAY SERVE AS CHEMO- AND RADIO-THERAPEUTIC SENSITIVE MARKERS

Drug resistance is a serious obstacle to effective cancer therapy [110]. The main mechanism of chemotherapy and radiotherapy is to induce DNA damage directly [5]. Cancer cells can also utilize the DNA repair machinery to process DNA lesions induced by DNA damaging agents in order to maintain cellular survival, which is therefore also an important mechanism of therapeutic resistance [111]. Patients with DDR defects are especially sensitive to these treatments.

Alkylating agents, including BCNU (1,3-bis(2-chloroethyl)-1-nitrosourea), procarbazine, streptozotocin, DTIC and TMZ, are among the most widely used chemotherapeutic drugs in human cancer [39]. Several alkylation sites in DNA have been identified as the targets of action of these compounds, and one of these sites is the O6 position of guanine [26]. Cytotoxic effects of alkylating agents are mediated primarily through methylation of O6-guanine, which leads to DNA double strand breaks and subsequent inhibition of DNA replication and apoptosis [26]. However, the toxicity of alkylating agents is reduced in the presence of MGMT by rapidly reversing the formation of adducts at the O6 position of guanine [26, 112]. For this reason, loss/reduced expression of MGMT can sensitize cancer cells to alkylating agents. In gliomas, enhanced sensitivity to alkylating agents was initially suggested in the subgroup of patients with reduced MGMT activity [41–43]. Since CpG island hypermethylation of MGMT is the major cause of loss of its expression in gliomas, MGMT methylated glioma patients are more sensitive to alkylating agents [19]. Thus, MGMT methylated patients acquired longer overall survival time after BCNU treatment [28, 107]. Similar results were obtained in colorectal cancer by treating MGMT methylated patients with TMZ [23]. Additional studies were reported in different cancer types (Table 1). Similar DNA damage effects can be mediated by radiotherapy through alkylation of O6-guanine. Methylation of MGMT sensitized glioma patients to radiotherapy or combined TMZ and radiotherapy [113, 114].

**Table 1:** Promoter region methylation of DNA damage repair genes

Repair system	Gene name	Tumor type application	Diagnosis and prognosis Marker	Sensitive marker	Ref
Direct reversal	MGMT	Gliomas, diffuse large B-cell lymphoma, NSCLC, colorectal, gastric, esophageal and head and neck cancers	Early detection, poor prognosis	TMZ, ACNU, BCNU, procarbazine sensitive	[19, 28–33]
BER	XRCC1	Gastric			[41]
	TDG	Myeloma			[42]
	MED1	Colorectal and ovarian			[43]
	OGG1	Breast and thyroid	Poor prognosis		[44, 45]
	WRN	NSCLC, colorectal, gastric, prostate, breast and thyroid cancers, chondro-sarcomas, non-Hodgkin lymphoma		Irinotecan sensitive	[47]
NER	XPC	Bladder cancer	Poor prognosis		[53]
	ERCC1	Glioma	Poor prognosis	Cisplatin sensitive	[54]
	hHR23B	Myeloma cells			[55]
	XPG	Ovarian		Nemorubicin resistance	[56]
MMR	MLH1	Colorectal, gastric, pancreatic, ovarian and breast cancers, NSCLC, ESCC, adult T-cell leukemia/lymphoma, oral squamous cell carcinoma, head and neck squamous cell carcinomas	Early detection, good prognosis	Carboplatin/cisplatin/TMZ/epirubicin/Oxaliplatin resistance Methotrexate sensitive	[60, 66–74]
	MSH2	HNPCC			[65]
	MSH3	Esophageal and gastric cancers			[144, 145]
Non-homologous end-joining	XRCC5	NSCLC			[80]
Homologous recombination	BRCA1	NSCLC, breast, ovarian, gastric, colorectal, bladder and pancreatic cancers	Diagnosis marker	Cisplatin, PARPi sensitive	[89, 93–97]
	BRCA2	NSCLC			[80]
	SRBC	Colorectal, lung, pancreatic, gastric and ovarian cancers		Oxaliplatin resistance	[146–149]
FA	FANCF	NSCLC, ovarian, breast, gastric, cervical and head and neck cancers		Cisplatin sensitive	[48, 89]
	FANCC	Head and neck squamous cell carcinoma, AML, ALL			[90, 91]
	FANCL	ALL			[91]
Cell cycle related	ATM	Breast cancer, head and neck squamous cell carcinomas		Radiotherapy sensitive	[150, 151]
	CHFR	NSCLC, breast, bladder, colorectal, gastric, nasopharyngeal, esophageal, cervical, hepatocellular and head and neck cancers	Poor prognosis	Docetaxel sensitive	[100]
	P16	Oral and oropharyngeal squamous cell carcinoma, esophageal, bladder and lung cancers	Poor prognosis	5-FU sensitive	[152–155]
	RASSF1A	Breast, ovarian, lung, prostate, colon, bladder and liver cancers	Early detection, Poor prognosis	Cisplatin and tamoxifen resistance	[156]
	CHK2	Glioma, NSCLC			[157, 158]
Genome “caretaker“	FHIT	Gastric, esophageal, cervical and breast cancers, NSCLC	Poor prognosis		[159–163]
Antioxidant	GPX3	Non-M3 acute myeloid leukemia, thyroid, liver and head and neck cancers	Poor prognosis	Cisplatin resistant	[164–167]
Detoxification of carcinogens	GST-Pi	Prostate, breast. kidney, liver and lung cancers	Poor prognosis	Doxorubicin sensitive	[168–170]

Repair of interstrand crosslinks induced by cisplatin leads to drug resistance. The expression of ERCC1 has been related to poor response and survival in cisplatin-treated NSCLC patients [1]. ERCC1 expression is a resistance marker of platinum-based systemic chemotherapy in human gastric and ovarian cancers [115, 116]. Methylation of ERCC1 is associated with cisplatin sensitivity in glioma cell lines [54]. BRCA1/2 deficiency results in cellular sensitivity to cisplatin. BRCA1 methylation has been found to predict significantly higher response rates to cisplatin treatment in patients with triple-negative breast cancer [117]. FANCF methylation is correlated with cisplatin sensitivity in ovarian cancer cells [118]. Demethylation of FANCF leads to acquired cisplatinum resistance [119]. CHFR methylation is a docetaxel sensitive marker in human esophageal, breast, gastric and other cancers [100]. MLH1 methylation is a resistance marker of oxaliplatin in human gastric cancer [67]. WRN methylation is an irinotecan sensitive marker in gastric cancer [120]. Methylation of ATM sensitized glioma and colorectal cancer cells to ionizing radiation therapy [121, 122]. As described above, disruption of DNA repair can promote genetic instability and further lead to tumorigenesis. On the other hand, defects in DNA damage repair sensitize cancer cells to DNA damaging agents. Thus, detection of genetic and epigenetic changes of DDR genes is reasonable before treatment.

## THERAPEUTIC TARGETING OF ABERRANT EPIGENETIC CHANGES IN DNA DAMAGE REPAIR GENES

### Demethylation of MMR genes sensitizes cancer cells to DNA damaging agents

MMR recognizes damaged DNA that was induced by DNA damaging agents, and it initiates the cell death machinery to trigger apoptosis [123, 124]. Disruption of MMR by DNA methylation failed to induce apoptosis [125]. In vitro studies have shown that treatment of MLH1-methylated colon cancer cell lines with the demethylating agent 5′-aza-2′-deoxycytidine (5-aza-dC) induced the expression of MLH1 and sensitized cancer cells to 5-fluorouracil (5-FU) [126]. In MLH1 methylated ovarian and colon cancer xenografts, cancer cells were sensitized to carboplatin, cisplatin, TMZ and epirubicin after re-expression of MLH1 by decitabine [127]. Another study demonstrated that demethylation of MLH1 was induced by low-dose 5-aza-dC and the sensitivity of radiotherapy was enhanced in colorectal cancer [128]. In a phase II clinical trial in ovarian cancer patients, decitabine induced MLH1 demethylation, increased the response rate of carboplatin and prolonged progression-free survival [129].

Recently, a novel DNA methylation inhibitor, SGI-110, resensitized a range of platinum-resistant ovarian cancer cells to cisplatin. SGI-110 alone or in combination with cisplatin enhanced antitumor effects in ovarian cancer xenografts [130]. While, SGI-110 is controversial in the treatment of gastric and ovarian cancer in combination with oxaliplatin in patients with MLH1 methylation [67, 131]. It has been reported that MMR proteins do not recognize the adducts formed by oxaliplatin [132]. XPG methylation leads to disruption of the NER system and is related to resistance of nemorubicin, a doxorubicin derivative. Restoration of XPG expression by demethylating agents restored the sensitivity of different cancer cells to nemorubicin [56].

### Synthetic lethality therapy in cancer patients with defects in DNA damage repair pathways induced by genetic or epigenetic abnormalities

There are a number of DNA repair pathways that protect cellular DNA from injury. “Cross-talk” between these pathways facilitates a unified guard for genomic integrity. The concept of synthetic lethality originates from studies in drosophila model systems in which a combination of mutations in two or more separate genes leads to cell death [133].

The strategy of synthetic lethality approaches is that defects in two different genes or pathways together result in cell death. While, independently defect does not affect viability [22]. Although only less than 10% of sporadic ovarian cancer and 5% of breast cancer harbored BRCA1 mutations, BRCA1 methylation is observed in approximately 11–14% of breast cancer and 5%–31% of ovarian cancer [93]. HR-defective BRCA1/2-deficient cell lines display dramatically increased sensitivity to inhibition of the single strand break repair enzyme PARP [81]. A phase II clinical trial demonstrated that monotherapy with the PARP inhibitor olaparib (AZD-2281; AstraZeneca) achieved encouraging response rates of 41% and 33% in patients with BRCA1- or BRCA2-mutated advanced breast and ovarian cancers, respectively [77]. Targeting RAD52 can also induce synthetic lethality in BRCA-deficient leukemias [134]. In addition, colorectal cancer cells with defects in MSH2 or MLH1 are sensitized to methotrexate [133, 135]. Many cancer cells acquire defects in a certain DNA repair pathway and become dependent on a compensatory mechanism in order to survive. Thus, pharmacological inhibition of the “backup” pathway in combination with DNA damage will selectively kill cancer cells but spare their normal counterparts [77].

### Deficiency of DNA damage repair caused by DNA mutation and methylation opens a new window for immune therapy in human cancers

DNA damage repair deficiency was found in various human cancers by gene mutation or promoter region hypermethylation, including MLH1, MSH2, MGMT, BRCA1, BRCA2 and others. Loss of efficiency of one or more DNA repair pathways can accelerate the rate of accumulation of additional mutations by 100–1,000 times [5]. Somatic mutations have the potential to encode “non-self” immunogenic antigens [136]. The programmed death 1 (PD-1) pathway is a negative feedback system that represses Th1 cytotoxic immune responses. It is up-regulated in many tumors and in their surrounding microenvironment [137, 138]. Blockade of this pathway with antibodies to PD-1 or its ligands has led to remarkable clinical responses in patients with many different types of cancer [136, 139–141]. Epigenetic inactivation of DNA damage repair genes could induce gene mutations and lead to alterations in the expression of tumor-associated self-antigens, therefore altering the antigenicity of the tumor. In a recent study, authors evaluated the clinical activity of pembrolizumab, a PD-1 inhibitor, in colorectal cancer patients with progressive metastatic cancer with or without mismatch-repair deficiency. For mismatch repair-deficient colorectal cancers, the immune-related objective response rate and immune-related progression-free survival rate were 40% and 78%, respectively. While, in mismatch repair-proficient colorectal cancer patients, the immune-related objective response rate and immune-related progression-free survival rate were 0% and 11%, respectively [136]. Whole-exome sequencing revealed that the somatic mutation number in mismatch repair-deficient tumors is much larger than in mismatch repair-proficient tumors, and high somatic mutation loads were associated with prolonged progression-free survival [136]. It was demonstrated in early studies that there is dense immune infiltration and a Th1-associated cytokine-rich environment in mismatch repair-deficient tumors [142]. The mismatch repair–deficient tumor microenvironment strongly expressed several immune checkpoint ligands, including PD-1, PD-L1, CTLA-4, LAG-3 and IDO, to protect the tumor cells from death. Thus, inhibition of these immune checkpoints can enhance T-cell responses and mediate effective antitumor activity in MMR defective cancers [143]. In addition to the MMR system, deficiencies of other DNA damage repair pathways play important roles in tumorigenesis. Thus, both mutational and epigenetic landscapes are necessary to stratify the tumor patients before immune therapy, especially for genes in DNA damage repair pathways.

## CONCLUSION &AMP; PERSPECTIVE

Precision medicine based on various ‘omics,’ such as genomics, transcriptomics and epigenomics, has already had an impact on the clinical care of cancer patients. Anticancer therapy could be selected on the basis of the molecular phenotype of the cancer. Targeting DNA repair proteins has significantly increased over the past decade. The impact of DNA repair on resistance to cisplatin is well documented in numerous cancers. Immune therapy has reached apparent efficiency in MMR defective cancers. MGMT methylation serves as a sensitive marker for alkylating agents in glioma patients, and CHFR methylation sensitizes esophageal cancer cells to docetaxel. Synthetic lethality is a more exciting approach in patients with DDR defects. PARP inhibitors are the most effective anticancer reagents in BRCA-deficient cancer cells.
